# Mechanisms of interpersonal sway synchrony and stability

**DOI:** 10.1098/rsif.2014.0751

**Published:** 2014-12-06

**Authors:** Raymond F. Reynolds, Callum J. Osler

**Affiliations:** 1 School of Sport, Exercise and Rehabilitation Sciences, University of Birmingham, Birmingham B15 2TT, UK; 2 Department of Life Sciences, University of Derby, Kedleston Road, Derby DE22 1GB, UK

**Keywords:** posture, interpersonal, feedback model

## Abstract

Here we explain the neural and mechanical mechanisms responsible for synchronizing sway and improving postural control during physical contact with another standing person. Postural control processes were modelled using an inverted pendulum under continuous feedback control. Interpersonal interactions were simulated either by coupling the sensory feedback loops or by physically coupling the pendulums with a damped spring. These simulations precisely recreated the timing and magnitude of sway interactions observed empirically. Effects of firmly grasping another person's shoulder were explained entirely by the mechanical linkage. This contrasted with light touch and/or visual contact, which were explained by a sensory weighting phenomenon; each person's estimate of upright was based on a weighted combination of veridical sensory feedback combined with a small contribution from their partner. Under these circumstances, the model predicted reductions in sway even without the need to distinguish between self and partner motion. Our findings explain the seemingly paradoxical observation that touching a swaying person can improve postural control.

## 1. Introduction

Cutaneous information has been shown to improve postural control [1–7]. Other than the sensation of force underfoot [8], a major source of cutaneous feedback arises from manual contact with our surroundings. Lightly touching an earth-fixed object can reduce sway as much as vision [5]. This occurs in the absence of mechanical stabilization, is abolished when the arm is anaesthetized [1] and has a delay characteristic of a sensorimotor feedback process [6]. So just like vision, proprioception and vestibular input, cutaneous information provides a powerful source of sensory feedback of bodily movement during stance.

Contact with *moving* objects also influences postural sway. For example, lightly touching a rhythmically oscillating surface induces body movement at the same frequency [3]. Unsurprisingly, this increases sway when compared with contact with a stationary reference point [9]. Perhaps more surprising is the recent observation that lightly touching another standing person can reduce sway, compared with no touch [10,11]. In this case, the moving ‘object’ is a human being, with the pseudorandom motion characteristics of postural sway. How this could be beneficial for balance, rather than deleterious, is open to question. Indeed, in a recent study which used a haptic robotic interface to mimic human sway, postural control when touching the device was no better than baseline sway, and was possibly worse [9].

This intriguing observation—that light touch with another standing person can *reduce* sway—raises many questions. What are the sensorimotor and/or mechanical mechanisms by which contact with another person can improve postural control? Is it necessary to differentiate self motion from partner motion to derive any benefit from interpersonal touch? Can physical contact with somebody *less* stable be beneficial? How is cutaneous information integrated with other sensory inputs? Here we address these questions by comparing empirical observations of interpersonal sway interactions with a postural control model. Varying levels of physical contact are combined with different visual conditions, in order to manipulate baseline sway. We then use a postural model based upon feedback control of an inverted pendulum to mimic human sway. Interpersonal interactions were recreated either by coupling the model feedback loops or by physically linking the pendulums, in order to represent sensory and mechanical coupling, respectively.

## 2. Material and methods

### 2.1. Participants

Sixteen right-handed volunteers gave informed consent to participate (eight males; aged 21–24). Volunteers were allocated to eight sex-matched pairs, matched approximately for height and weight. Although volunteers were acquainted with each other prior to the start of the experiment, none were in a relationship.

### 2.2. Procedure and measurements

**Figure 1.**
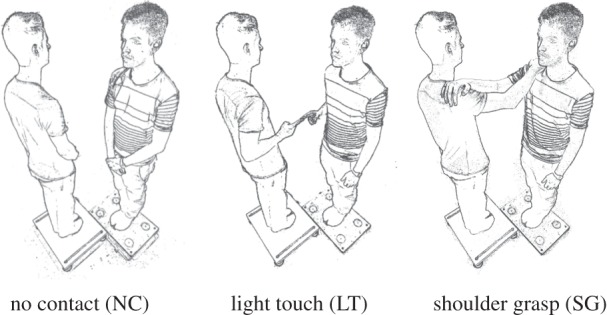
Experimental conditions. Each participant stood on a separate force platform. There were three touch conditions (shown in figure) and four visual conditions (EC, EO, AS1 and AS2) producing a total of 12 conditions. During the LT condition a FSR was placed between participants' fingertips to monitor contact force.

Each volunteer stood barefoot with feet together on a separate force platform (figure 1; Kistler 9281B and 9260AA6; Kistler Instrument Co., Winterthur, Switzerland). The force platform configuration allowed participants to stand facing each other, but offset such that their right shoulders were aligned. The medio-lateral offset was 30 cm (between mid-stance location), and anterior–posterior offset was 45 cm. This made for a precisely symmetrical postural arrangement allowing physical interaction between the right arms. Three conditions of varying contact were used: no contact (NC), firm shoulder grasp (SG) and light finger touch (LT). LT was maintained between the distal phalanges of the outstretched index fingers. A wafer-thin force sensitive resistor (FSR) was placed between the fingers to monitor contact force (Interlink Electronics Inc., Camarillo, CA, USA). Subjects were allowed a trial to maintain force below 1 N. Vision was systematically manipulated in four conditions: (i) both eyes open (EO); (ii) both eyes closed (EC); (iii) subject A eyes open/B eyes closed (AS1); and (iv) subject A eyes closed/B eyes open (AS2). When vision was available, subjects were instructed to look directly ahead at a point marked on the laboratory wall. Hence, given the stance geometry, the angle between gaze direction and the centre of the partner's head was approximately 34°.

The experiment involved a 3 × 4 factorial design (3 contact × 4 vision conditions), leading to a total of 12 conditions. Each condition was studied during two 90 s trials of quiet standing (i.e. 24 trials in total). Trial order was randomized. To abolish any potential bias associated with standing in a particular configuration within the room, or on a particular force platform, subjects swapped location between trials. Force sensitive resistive and ground reaction forces were sampled at 1 kHz (NI PCI-6229 DAQ).

### 2.3. Analysis

All data were analysed using Matlab (Mathworks, USA). For each subject pair, data from both subjects were combined to remove any effects caused by individual differences. For example, in the AS1/LT condition, when subject A had his/her eyes open, these data were combined with those of subject B during the trial when *their* eyes were open. This analysis ensured that we only measured the *interaction* between subjects, unaffected by possible differences in height, weight, balance ability, etc.

As the FSR is nonlinear, its output was first transformed using a calibration curve plotting voltage against known forces. This was achieved by placing small weights over the FSR ensuring that its full surface area was activated. Mean touch force was then calculated for each light contact trial. Centre of pressure (COP) was calculated from ground reaction forces, low-pass filtered (10 Hz, fourth order, zero-phase shift, Butterworth) and differentiated to give COP velocity (COPv). We analysed COP *velocity*, rather than position, for two reasons. Firstly, it is a more reliable [12] and direct [13] measure of postural stability than COP position, demonstrating stronger correlation with fall risk for example [14]. Secondly, COP position is a non-stationary signal [15,16] which renders it inappropriate for spectral or cross-correlation analysis [17]. Differentiating non-stationary signals is a recognized method for rendering them stationary [17]. Root mean square (RMS) COPv was used as the primary measure of postural sway (RMS COP *position* data are included in the electronic supplementary material). To characterize the timing and amplitude of interactions between subjects, unbiased cross-correlations were computed from COPv traces from each subject using the Matlab XCOV function with the ‘coeff’ option activated. This normalizes the sequences such that the auto-correlation function at zero lag is precisely 1 for each signal. The resulting cross-correlation values vary between 1 and −1, representing a perfect positive and negative correlation, respectively. The asymmetric visual conditions (AS1 and AS2) were combined for this analysis (reducing the conditions from 12 to 9). We also analysed coherence between subjects, as a measure of coupling in the frequency domain. These data, along with associated methodological details, are included in the electronic supplementary material.

### 2.4. Modelling

**Figure 2.**
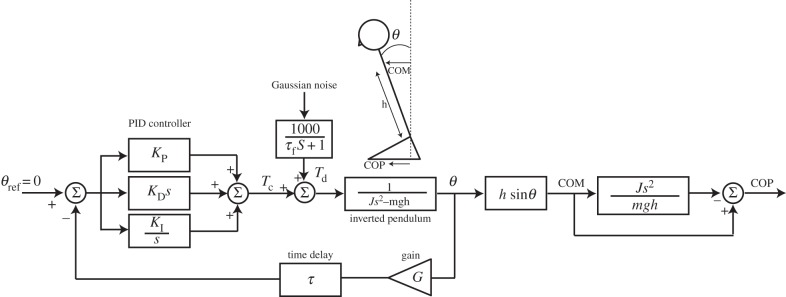
Postural control model. The model consists of an inverted pendulum under PID control, based upon Peterka [19]. The parameters *K*_P_, *K*_I_ and *K*_D_ define the PID constants, respectively. Sway was generated by adding a disturbance signal (*T*_d_) to the control torque (*T*_c_). This consisted of filtered Gaussian noise. Pendulum angle (*θ*) was processed to derive centre of mass (COM) and COP. COP was subsequently differentiated to obtain COPv for comparison with the empirical data. Pendulum moment of inertia is denoted by *J*, and *s* depicts the Laplacian operator. Parameter values are depicted in table 1. For further details, see Peterka [19].

Simulink was used to develop a postural model consisting of an inverted pendulum under proportional–integrative–derivative (PID) feedback control, with a delay representing sensorimotor processing time (figure 2). The input to the model is an error signal based upon the difference between a desired and actual position (body position in this case). The output signal (ankle torque in this case) is a corrective signal intended to reduce this difference. The output signal is then applied to the inverted pendulum, correcting its position, thus generating a new feedback signal. So the model operates by tuning the required torque signal to stabilize the pendulum, based upon feedback of its position. Such a model has previously been shown to successfully recreate the statistical characteristics of postural sway [18–20]. The pendulum is destabilized by applying noise to the torque signal. The physical properties of the pendulum were fixed, as were the proportional, integrative and derivative values of the PID controller, based upon values derived by Peterka [19] (table 1). All other parameters were varied as described below.

**Table 1.** Fixed parameter values. PID controller values were fixed, as were mechanical properties of the inverted pendulum and the filter time constant. These values are taken from Peterka [19]. All other parameters were allowed to vary during the model optimization procedure.

parameter	value
*K*_P_	19.5 N m deg^−1^
*K*_D_	4.5 N m s deg^−1^
*K*_I_	0.25 N m s^−1^
*J*	66 kg m^2^
*m*	76 kg
*h*	0.87 m
*τ*_f_	80 s

**Figure 3.**
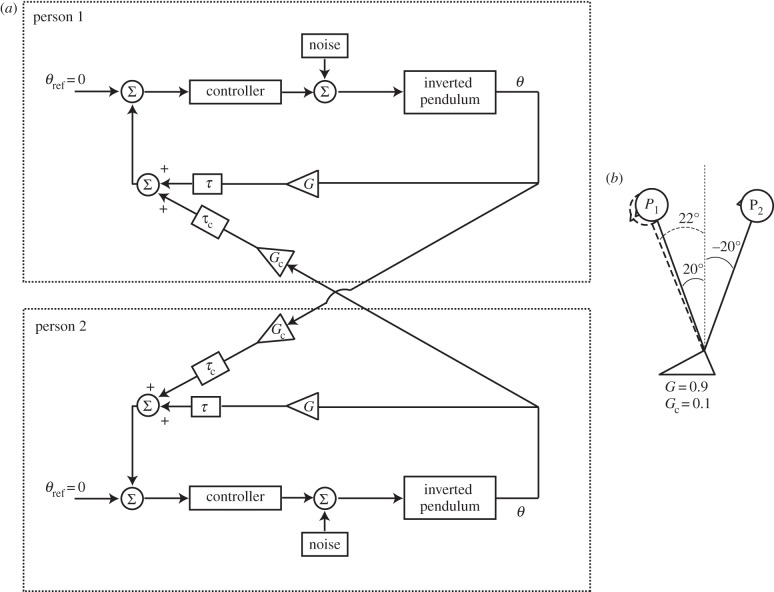
Coupling the model feedback loops. (*a*) A simplified version of the model, adapted from figure 2, is duplicated above to represent two people interacting. Feedback loops are coupled together. The estimate of pendulum position which feeds into the PID controller is a weighted combination of ‘self’ and ‘partner’. The weightings are determined by the gain functions *G* and *G*_c_. As *G*_c_ increases and *G* is reduced, the estimation becomes more reliant upon partner feedback. This principle is depicted in (*b*). Person 1's estimate of body position (dotted figure) is a weighted combination of veridical feedback combined with a small contribution from person 2. Assuming gains of 0.9(*G*) and 0.1(*G*_c_), this means that person 1's estimate of body position is (0.9 × 20) + (0.1 × 40) = 22°. An implicit assumption is that each person assumes the other to be a fixed vertical reference point.

**Figure 4.**
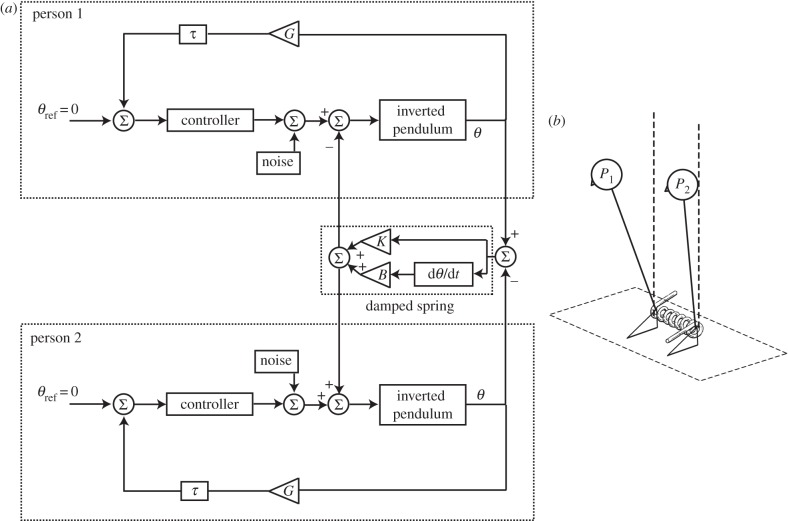
Physically coupling the pendulums. (*a*,*b*) The two model subjects are physically coupled together by a damped spring. This is depicted schematically in (*b*) by the torsional spring. As one pendulum moves, it will exert torque upon the other, and vice versa. The torque exerted by the spring is a function of the difference in pendulum positions. *K* and *B* represent stiffness and damping, respectively. Although omitted for clarity, when physical coupling was engaged it was always combined with the feedback loop coupling depicted in figure 3.

To mimic interpersonal interaction, two such models were coupled together. To simulate the LT condition, as well as visual interactions, the feedback loops were coupled (figure 3). The consequence of this connection is that the estimate of pendulum position feeding into the PID controller is a combination of veridical feedback from ‘self’, plus feedback from the ‘partner’ pendulum. By varying the gain of each of these channels (self and partner feedback), the relative contribution of the two channels to the estimate of pendulum position could be varied. An implicit assumption of this arrangement is that the ‘partner’ pendulum is assumed to be a fixed reference point. This estimation process is schematically represented within figure 3. To mimic the SG condition, the two pendulums were directly connected via a damped spring (figure 4). This causes motion of one pendulum to exert torque upon the other, and vice versa. When physical coupling was engaged, it was always combined with feedback coupling. The random torque perturbation signal was generated using a different starting seed for the two model subjects, but had identical statistical properties, as determined by the filter (figure 2).

COPv traces were generated from the model (figure 2). COPv–COPv cross-correlations were then performed using the method described above, for direct comparison with the empirical data. Simulink Design Optimization toolbox was used to vary model parameters until the best match was formed between the empirical and model cross-correlations. Optimized parameters were feedback gains and delays, and spring stiffness and damping. The only constraint applied was an upper limit of 1 for gain values. For the asymmetrical vision condition (AS), separate values for gain/delay were applied to each (model) subject, effectively doubling the number of parameters. The optimization procedure was reliable, that is, it always converged on the same solution despite changing parameter starting values by ±30% from the converged value.

### 2.5. Statistical comparisons

For analysis of empirical sway data, repeated-measures ANOVA was used to check for significant main effects of contact and vision, as well as interactions. ANOVA was also used to determine any effect of visual condition upon mean touch force during the LT condition. When twin peaks occurred in a cross-correlation, the *t*-test was used to determine any difference in the magnitude and timing of the peaks. Similarity between empirical and model cross-correlations was determined by Pearson correlations. *p* < 0.05 was considered significant for all tests.

## 3. Results

### 3.1. Touch force

During the LT condition mean touch force was approximately 0.5 N and was not influenced by vision (mean ± s.d.: 0.47 ± 0.10 N (EC), 0.48 ± 0.07 N (AS) and 0.51 ± 0.12 N (EO); *F*_2,14_ = 1.2; *p* = 0.33).

### 3.2. Sway

**Figure 5.**
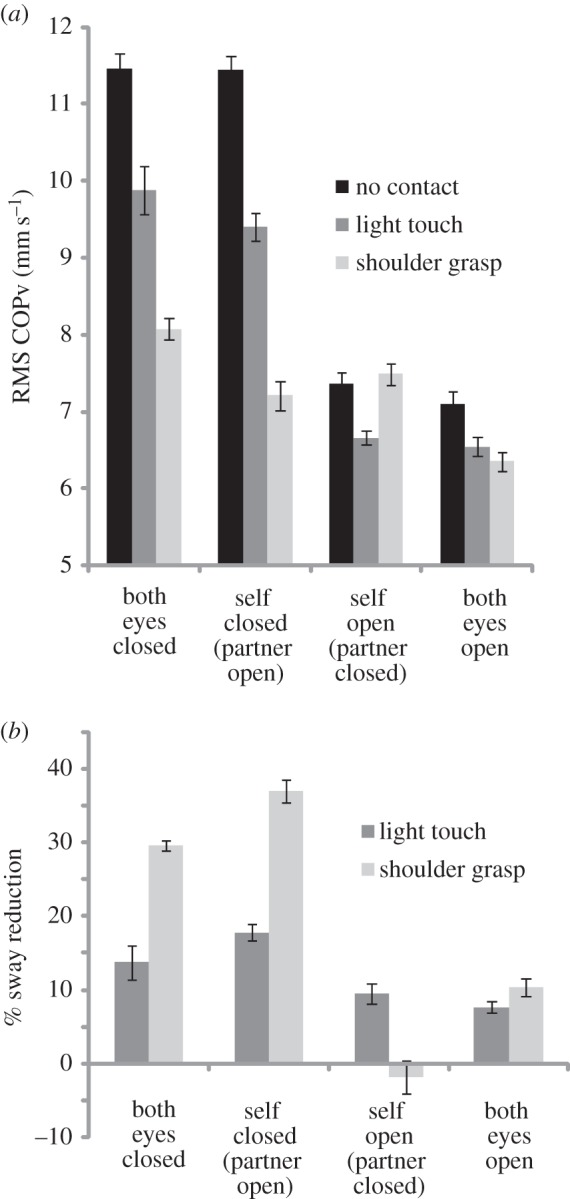
Effects of interpersonal touch and vision upon sway. Sagital COPv is shown in (*a*) for all conditions. Percentage changes in sway relative to the NC condition are shown in (*b*). Bars show s.e.m.

RMS sagittal COPv is shown in figure 5*a* for all conditions. Most sway occurred with the eyes closed during the NC condition. As expected, opening the eyes reduced sway. LT also reduced sway, and SG reduced it further. These observations were confirmed by significant main effects of contact (*F*_2,14_ = 354; *p* < 0.001) and visual condition (*F*_3,21_ = 845; *p* < 0.001). Furthermore, an interaction between these factors confirms that physical contact had the greatest stabilizing influence when vision was absent (*F*_6,42_ = 108; *p* < 0.001). Significant main effects of vision and contact, and the interaction, also occurred for RMS sagittal COP *position* (included in the electronic supplementary material).

To determine changes *relative* to the NC baseline condition, percentage sway reduction was also calculated (figure 5*b*). The significant main effects of contact and vision, plus the interaction, all persisted after this normalization procedure (*p* < 0.001). Both types of contact had the greatest stabilizing effect when a subject had their eyes closed and their partner had their eyes open. In this condition, SG and LT reduced sway by 37% and 18%, respectively. The only condition where physical contact did *not* reduce sway was when a subject with eyes open grasped the shoulder of a partner with eyes closed.

### 3.3. Cross-correlations

**Figure 6.**
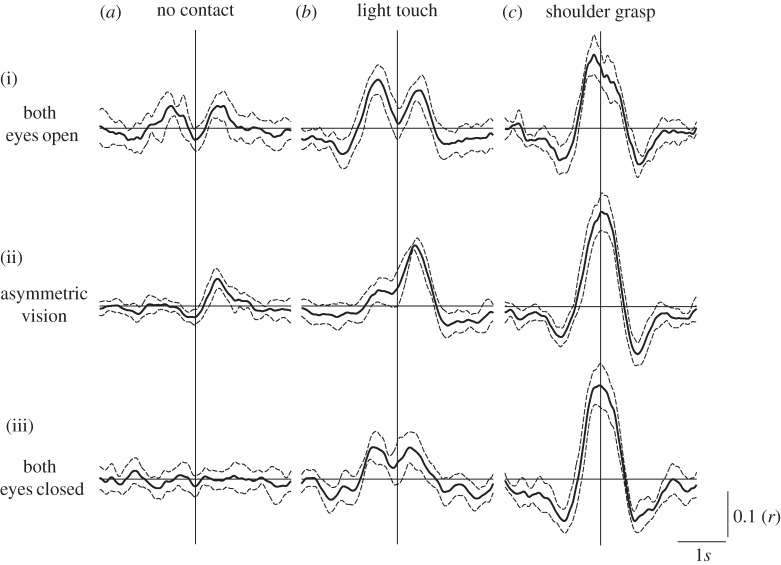
Sway cross-correlations. Sagittal COPv traces from each subject pair were cross-correlated. Traces depict mean *r* values ±95% confidence intervals. Vertical and horizontal lines depict zero-lag and zero-correlation values, respectively. ‘Asymmetric vision’ refers to the condition in which one subject's eyes were open and their partner's closed. In this condition, significant correlations at positive lag reflect the ‘blind’ subject leading the sighted.

Significant sway coupling was evident by deviation of the cross-correlation 95% confidence limits from zero (figure 6). This occurred for all conditions except EC/NC (figure 6*a*(iii)). In the EC/LT condition (figure 6*b*(iii)) two peaks are visible either side of zero, indicating that each subject followed the movement of their partner with a delay. When both subjects opened their eyes, these peaks become more prominent (figure 6*b*(i)). Vision *alone* also produced significant correlations with a similar pattern, albeit of smaller magnitude (figure 6*a*(i)). In the asymmetric visual conditions (figure 6*b*(i)(ii)(iii)), a peak at positive lag indicates that the ‘blind’ subject leads their ‘sighted’ partner. However, a smaller, but significant peak at negative lag was also identifiable in the AS/LT condition. The SG condition produced a very different pattern, with a large prominent peak around lag zero regardless of visual condition (figure 6*c*(i)(ii)(iii)).

**Figure 7.**
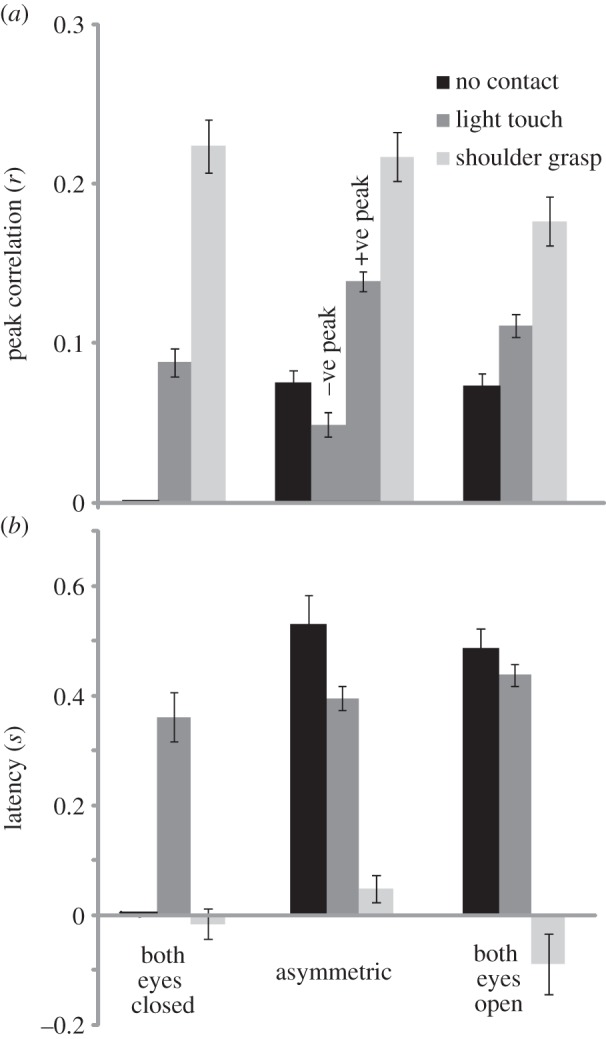
(*a*,*b*) Cross-correlation peak values and latencies. Average maxima and latencies of COPv–COPv cross-correlations.

Magnitudes and latencies of cross-correlation peaks are shown in figure 7. When twin peaks occurred, the data from both peaks were combined unless there was a significant difference between them (i.e. *p* < 0.05; *t*-test). This only occurred for the *magnitude* of the LT/AS condition (i.e. figure 6*b*(ii); *t* = 16.8; *p* < 0.001). Hence both peaks (negative and positive) are represented separately in figure 7*a* for this condition. SG consistently produced the largest correlations (0.18–0.22), with latencies that were not significantly different from zero (figure 7*b*; *t* ≤ 1.89; *p* ≥ 0.099). Vision alone (EO/NC or AS/NC) produced significant correlation magnitudes, but with longer latencies than seen during light contact alone (485 and 361 ms for EO/NC and EC/LT conditions, respectively; *t* = 2.55; *p* = 0.038).

### 3.4. Postural model

**Figure 8.**
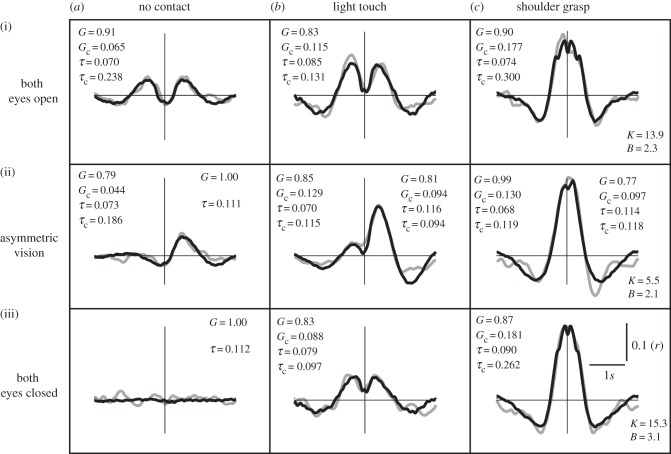
(*a*–*c*) Model sway cross-correlations. A postural feedback control model was used to generate sway cross-correlations (black) for direct comparison against empirical data (grey). Insets are parameter values derived from the model optimization procedure. *G* = gain; *G*_c_ = coupling gain; *τ* = feedback delay (s); *τ*_c_ = coupling feedback delay(s). For asymmetric conditions (*a*(ii),*b*(ii),*c*(ii)), separate values were ascribed to each model subject. For the shoulder contact condition (*c*), the physical interaction between subjects was modelled by a damped spring with the following parameters: *K* = stiffness (Nm deg^−1^); *B* = damping (Nm deg^−1^ s^−1^).

To mimic LT and visual coupling, the model feedback loops were coupled together (figure 3). For the SG condition, this was combined with a damped spring coupling (figure 4). Feedback gains and delays, and spring damping and stiffness were varied until the best match was formed between the empirical and model cross-correlations. The resulting comparison can be seen in figure 8. The model successfully recreated the overall shape, magnitude and timing of the cross-correlations for all conditions (correlation between model and empirical cross-correlations; *r*^2^ = 0.78–0.97; *p* < 0.0001). The model parameter estimates for gain (*G*) varied between 0.77 and 1, while coupling gain (*G*_c_) varied between 0.044 and 0.181. For the SG condition, spring stiffness varied between 5.5 and 15.3 Nm deg^−1^, while damping was 2.1–3.1 Nm deg^−1^ s^−1^. Sensorimotor delay estimates were 68–181 ms (*τ*) and 97–300 ms (*τ*_c_). A comparison of the EO/NC versus EC/LT conditions shows that the estimate of coupling delay (*τ*_c_) was greater for vision alone (238 ms) than for touch alone (97 ms).

### 3.5. Effects of varying model parameters

To determine how model parameters affected sway we compared coupled versus uncoupled sway while systematically varying gains, delays, stiffness and damping. These parameters were varied to span the range of values derived in figure 8 and were varied in an identical way for both model subjects, thus maintaining a symmetrical relationship for the pair.

#### 3.5.1. Gain

**Figure 9.**
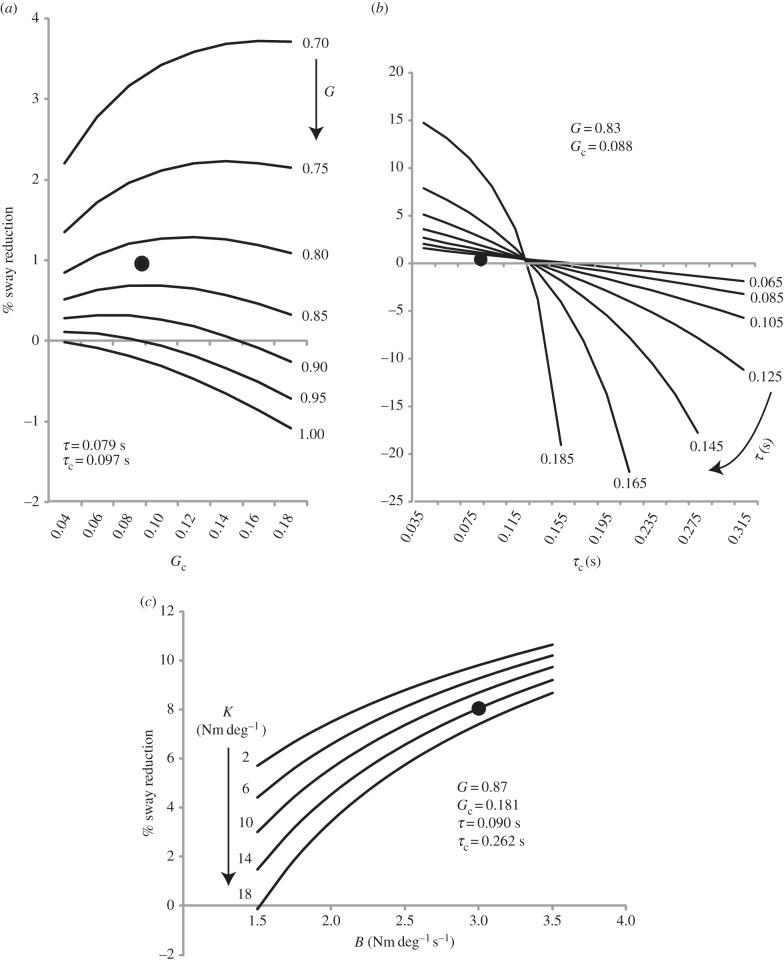
Effect of model parameter variation upon sway reduction. Model parameters were systematically varied to determine their effect upon sway. Sway reduction was calculated as the percentage change between coupled and uncoupled models. Positive values indicate that coupling reduced sway. (*a*) The effect of varying gains while delays were kept constant. (*b*) The effect of varying delays while gains were kept constant. (*c*) The effect of varying stiffness and damping when the models were coupled by a damped spring (in addition to coupling the feedback loops). Black dots depict percentage sway reduction when parameters were fixed to those values derived by the optimization procedure (figure 8). *G* = gain; *G*_c_ = coupling gain; *τ* = delay (s); *τ*_c_ = coupling delay (s); *K* = stiffness; *B* = damping.

Figure 9*a* shows the influence of varying gains upon the sway-reducing effect of coupling the feedback loops (i.e. the coupling depicted in figure 3). Delays were fixed according to parameters derived from the LT/EC condition (*τ* = 0.079 s and *τ*_c_ = 0.097 s). It can be seen that, with parameters set to those values derived from the optimization procedure, the model predicts a reduction in sway caused by coupling (see inset black dot). As gain (*G*) was systematically increased from 0.7 to 1, the sway-reducing benefit of coupling decreased. At the highest value of gain (*G* = 1), coupling actually caused an *increase* in sway. Coupling gain (*G*_c_) interacted with this effect; there was an optimal *G*_c_ value for sway reduction at each value of *G*. For example, at *G* = 0.8 the greatest sway reduction occurred at a *G*_c_ of 0.12.

#### 3.5.2. Delay

Effects of delay (*τ*) and coupling delay (*τ*_c_) are shown in figure 9*b* (gains fixed at *G* = 0.83 and *G*_c_ = 0.088). There was most to be gained from coupling when *τ*_c_ was low and *τ* was high (i.e. towards top left of figure 9*b*). As *τ*_c_ increases beyond a critical point (approx. 125 ms), coupling becomes deleterious (i.e. towards bottom right of figure 9*b*). Furthermore, an interaction between *τ* and *τ*_c_ is evident.

#### 3.5.3. Stiffness and damping

Figure 9*c* shows the influence of stiffness (*K*) and damping (*B*) upon the sway-reducing effect of physical coupling via the damped spring (depicted in figure 4). Gains and delays were kept constant at those values derived for the EC/SG condition. *K* and *B* were varied across a range of values derived from the empirical matching process depicted in figure 8. The introduction of the damped spring produced a reduction in sway across almost all tested values, with the largest reduction being 11% (*K* = 2 Nm deg^−1^; *B* = 3.5 Nm deg^−1^ s^−1^). *K* and *B* had opposing effects, i.e. *increasing B* or *decreasing K* caused more sway reduction. Changing *K* had a relatively small influence compared with changing *B*, which may explain the fairly large range of stiffness values derived by the model optimization process (5.5–15.3 Nm deg^−1^ compared with 2.1–3.1 Nm deg^−1^ s^−1^ for damping; see figure 8*c*(i)(ii)(iii)).

### 3.6. Effect of model asymmetry

**Figure 10.**
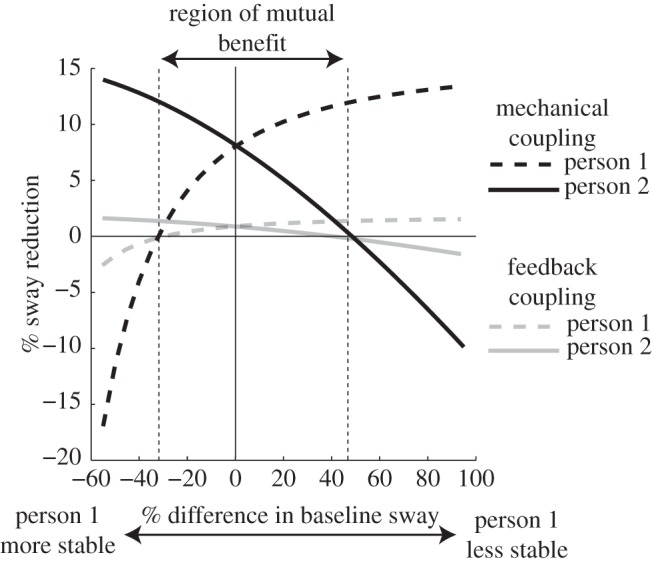
Effect of different baseline sway upon benefits of coupling. The baseline (uncoupled) sway of person 1 was systematically modulated by changing their noise gain while person 2's baseline sway was kept constant. The beneficial effect of coupling was then measured as the percentage change between coupled and uncoupled sway, for each model person. The region of mutual benefit refers to the area where both persons exhibit a reduction in sway, despite differences in baseline stability.

The empirical data from figure 5 show that interpersonal contact was not always beneficial when there were differences between subjects in terms of their baseline (NC) sway. This was particularly apparent during the AS/SG condition. To determine if our model captures this effect, we induced asymmetry in baseline sway between model subjects by altering the magnitude of Gaussian noise applied to the torque signal (ordinarily fixed at 1000 in figure 2). Figure 10 shows the effect of this asymmetry upon the beneficial effect of feedback and mechanical coupling. The figure demonstrates greatest sway reduction when coupling with a more stable person. However, there is a considerable region of difference where both model subjects benefit from coupling, despite the considerable differences in baseline sway. Once a threshold is reached (−35% to +45% difference), coupling is only beneficial for the less stable subject with no benefit for the more stable subject, becoming detrimental to the latter beyond this threshold. This compares favourably to the empirical data in figure 5; the effect of vision was to reduce sway by approximately 36% (11.45 versus 7.36 mm s^−1^). Under the asymmetric condition, SG produced considerable benefit for the eyes closed subject, with no discernible benefit for their more stable eyes open partner.

## 4. Discussion

Our results explain the basis of the recently observed phenomenon in which physical contact with another swaying person is beneficial for postural stability [10,11]. A relatively simple postural control model accurately captured the interactions between two standing persons. This allowed for identification of the sensory and mechanical mechanisms underlying these interactions. It also explains how physical contact with another person can reduce sway even when both persons have equal baseline sway, and without the need for the nervous system to explicitly distinguish between self and partner motion.

Our empirical observations confirm that physical contact with another person improves postural control. SG and light contact reduced sway by up to 37% and 18%, respectively, which tallies well with previous findings (13% effect of LT in 10, up to approximately 40% in 11 depending on stance). Our results extend these observations. Firstly, the sway-reducing effect of physical contact interacted with vision. This interaction was due to the beneficial effect of contact being greatest when closing the eyes reduced baseline stability. Secondly, individuals had the most to benefit when making contact with somebody stabilized by vision. Greatest sway reduction occurred when a person with their eyes closed grasped the shoulder of their eyes open partner. This complements the findings of Johanssen *et al.* [11], who showed a similar effect caused by stance differences between subjects. Nevertheless, we did observe small reductions in sway for eyes open participants making LT with an eyes closed partner. This indicates that interpersonal contact can be beneficial even when making contact with a *less* stable partner, albeit to a lesser extent. Presumably this benefit disappears when the stability differential reaches some threshold, as predicted by the model. In contrast to the sway reductions we observed here, Wing *et al.* [9] noted an *increase* in sway when touching a haptic interface replaying recorded human sway. There may be a number of reasons for this discrepancy. Firstly, in contrast to a real person, the haptic interface produced a pre-programmed ballistic motion, with no possibility of two-way interaction. Secondly, the stiffness characteristics of the interface may differ from a human arm. Lastly, there may be cognitive or psychological factors when interacting with a known unstable object, which could alter tactile weighting.

In addition to improving postural stability, physical contact caused sway entrainment, as revealed by the COPv–COPv cross-correlation. The precise timing of this coupling varied according to contact type. LT alone produced twin peaks with a delay around ± 380 ms. This delay suggests a sensory feedback control mechanism, whereby each person interprets changing fingertip force as self motion, and produces a compensatory sway response. Vision *alone* produced a very similar pattern to LT, but with an additional delay of approximately 100 ms. This is probably due to known differences in cutaneous and visual processing time [21,22] (although see [23]), but might also reflect differences in the representation of body position, velocity or acceleration between sensory modalities. To our knowledge, this is the first demonstration of interpersonal sway coupling by vision alone. This occurred despite only peripheral vision of one's partner, and full visual feedback from the static laboratory surroundings. It is difficult to estimate the consequence of interpersonal visual coupling upon sway magnitude since, in our experimental set-up, opening the eyes simultaneously provided feedback from both the other person *and* the laboratory. The latter probably dominates in terms of sway reduction. But in situations where interpersonal vision dominates, such as crowded outdoor spaces, this coupling may have a strong influence on postural sway. Reliance upon vision could be highly destabilizing in such situations, particularly if other sensory channels are compromised by age or pathology. During the asymmetrical condition, when only one subject had vision, the ‘blind’ subject led the sighted. This is shown by the cross-correlation peak at positive lag (figure 6). Given that the baseline sway of the eyes closed subject is greater than their eyes open partner, this influence is unlikely to benefit the latter. But, as stated above, vision provided ample beneficial feedback from the laboratory, which probably dominates over peripheral vision of an unstable partner. In contrast to vision and/or LT, SG resulted in a cross-correlation with a single large peak around zero lag, consistent with a direct physical link. Similar to LT, SG would also provide cutaneous feedback, in addition to mechanical effects. However, the consistent zero-lag peak suggests that any such effect was swamped by the mechanical influence. Furthermore, the cross-correlation was unaffected by visual conditions. Taken together, these results are consistent with two mechanisms: a sensorimotor feedback delay process for vision and LT and direct mechanical coupling for SG.

These mechanisms are corroborated by the model, which reproduced the empirical observations with remarkable fidelity (figure 8). Each person was represented by a PID feedback-controlled inverted pendulum, with physiologically plausible delays in the feedback loop. This type of model has previously been shown to accurately reproduce many characteristics of standing posture, including statistical properties of the COP [19] and sensory reweighting phenomena [20]. By coupling two such models together, we successfully recreated the timing of sway interactions. The estimated feedback delays (*τ* and *τ*_c_) were inevitably lower than the delays derived from the cross-correlation peaks, as they provide an estimate of sensorimotor delay unaffected by the mechanics of the inverted pendulum. The strongest coupling was achieved by physically linking the pendulums with a damped spring. This recreated the zero-lag response observed during SG, consistent with this effect being predominantly mechanical in nature. By contrast, the visual and tactile interactions were simulated by coupling the sensory feedback loops. This recreated the twin peaks of the cross-correlation function. One possible interpretation of twin peaks might be that one person acts as a sway leader and the other as a follower, periodically switching roles. Previous research has suggested that such relationships may occur during interpersonal LT and that the tendency to follow another person (or not) may depend upon balance expertise [24,25]. However, the model we employed was a linear time-invariant system, in which all parameters were kept constant during each simulation. It did not include any switching mechanisms or other time-dependent changes in behaviour which might render one person a follower and the other a leader. Under these circumstances, and even when both model subjects were identical, the simulations resulted in a twin-peaked cross-correlation. While this is not direct proof against a leader–follower relationship, it shows that it is unnecessary to invoke such a mechanism to explain the behaviour.

By coupling the sensory feedback loops, the model implies that each person's estimate of body position is based largely upon veridical feedback of body orientation, and partly upon the position of their partner. Physiological sources of veridical feedback would include ankle joint proprioception, vestibular input and vision of the laboratory. Feedback of partner body position would arise from fingertip force and peripheral vision of the other person. An implicit assumption of this sensory coupling is that each person assumes their partner to be a fixed reference point. That is, any change in fingertip force (or visual motion of one's partner) is interpreted as self motion. Despite this limitation, the model consistently predicted a reduction in sway (black dots in figure 9), in addition to recreating the response timing. This suggests that interpersonal contact is beneficial even when the central nervous system (CNS) cannot distinguish self motion (reafference) from partner motion (exafference). Of course, the CNS is capable of distinguishing reafference from exafference during voluntary movement [26], but whether this is true for the small involuntary movements of postural sway is less certain. If so, it would allow people to distinguish between fingertip forces caused by self versus partner motion, which, in turn, may improve postural control. Such a mechanism may explain why the empirically observed sway reductions were greater than those produced by the model. In addition to recreating the timing of the sway relationship and producing a reduction in sway, the model also predicted the effect of differences in baseline sway between participants. When one (model) person was sufficiently more stable than another, coupling only benefited the unstable person. However, there is a considerable region of difference where the model predicted benefits for *both* participants. This reflects the behavioural data which demonstrated some reduction in sway even when contacting a less stable partner.

Our model inevitably involved some assumptions and simplifications. For example, we used a damped spring to mimic the physical linkage produced by SG. This implies that the arms were entirely passive, whereas it is possible that some active forces were continuously generated by the elbow flexors and extensors. Furthermore, we simplified the postural control process with a linear system, in which sensory feedback gains were constant over time. Responses to sensory perturbations suggest that feedback gains change with stimulus amplitude [27,28]. However, such nonlinearity may be less relevant for the smaller range of sway seen during natural unperturbed standing. Lastly, when coupling the feedback loops our model did not explicitly represent the form of sensory feedback available to each person, in terms of fingertip force or visual input. Despite these simplifications, the model explained the data with remarkable precision, suggesting that our parsimonious approach captured the essence of the underlying mechanisms.

## Supplementary Material

Supplementary data
